# Protective Effect of Paeoniae Radix Alba Carbonisata on Hepatic Amyloidosis by Regulating Calcium Homeostasis

**DOI:** 10.3390/ijms27062582

**Published:** 2026-03-11

**Authors:** Gangqiang Liu, Zerui Wang, Huihui Xu, Jinyu Jia, Zhong Xue, Wei Ge, Xueqing Ji, Lijian Cui, Yun Huang

**Affiliations:** 1 School of Pharmacy, Hebei Medical University, Shijiazhuang 050017, China; 15939385272@163.com (G.L.); 15032719621@163.com (Z.W.); xhh2685551123@163.com (H.X.); jiajinyu546654@163.com (J.J.); 19000980@hebmu.edu.cn (Z.X.); gg20020415@163.com (W.G.); 15729589562@163.com (X.J.); 2 Experiment Center, Hebei University of Chinese Medicine, Shijiazhuang 050020, China

**Keywords:** Paeoniae Radix Alba Carbonisata, hepatic amyloidosis, calcium homeostasis, cGMP/PKG/ATP2A1 signaling axis, inflammation

## Abstract

Paeoniae Radix Alba Carbonisata (PRAC), carbonized decoction pieces of the traditional Chinese medicine Paeoniae Radix Alba, has been used in clinical practice for hepatoprotective purposes. Hepatic amyloidosis (HA), a severe complication of systemic AA amyloidosis, is characterized by the deposition of fibrillar amyloid proteins leading to progressive hepatic dysfunction. However, its role in HA remains unclear. Amyloid lysozyme (LYSO-6) was used to induce the NCTC1469 cell injury model and the HA mouse model. The effects of PRAC extract (PRAC-E) on liver injury were then evaluated using biochemical assays, enzyme-linked immunosorbent assay (ELISA), Congo red (CR) staining, Hematoxylin and Eosin (H&E) staining, and immunohistochemical staining. Liver transcriptomics combined with Western blotting was used to analyze the expression levels of key proteins in the cGMP/PKG/ATP2A1 signaling axis. UHPLC-Q-Exactive Orbitrap MS combined with network pharmacology was used to characterize the chemical components of PRAC-E and identify its core active constituents against HA. Quantitative analysis of core components was performed by UHPLC-QTRAP-MS/MS. Molecular docking predicted the binding stability of core components and key targets. The results showed that PRAC-E significantly alleviated HA. Collectively, PRAC-E restored calcium pump activity, corrected calcium homeostasis imbalance, reduced inflammatory factor levels, regulated Phosphodiesterase 5A (PDE5A), and activated the cGMP/PKG/ATP2A1 signaling axis. The main components of PRAC-E were phenolic acids, terpenoids, and flavonoids. Among these, six core components (SCCs) related to HA were Gallate (16.96 mg/g), Paeoniflorin (14.27 mg/g), Albiflorin (7.20 mg/g), Benzoyl paeoniflorin (5.33 mg/g), Methyl gallate (0.78 mg/g), and Catechin (0.09 mg/g). Molecular docking analysis demonstrated that SCCs formed stable complexes (∆*G* ≤ −6.2 kcal/mol) with ATP2A1.

## 1. Introduction

Amyloidosis is a protein misfolding disease caused by the autologous proteins that deposit as amyloid fibrils in tissues [1]. These fibrous aggregates are deposited extracellularly in many organs, leading to organ dysfunction and death [2]. An evaluation of 3987 amyloidosis patients from major referral centers in the United States reported a 2.5-fold annual increase in amyloidosis cases during the survey period [3]. The liver is a common site of involvement, involved in 60–90% of cases at autopsy [4]. Amyloid A (AA) amyloidosis is the main form of secondary hepatic amyloidosis (HA) [5]. Interleukin-6 (IL-6), Interleukin-8 (IL-8), and Tumor necrosis factor alpha (TNF-α) are thought to be the key factors promoting the production of AA [6,7]. Serum amyloid A (SAA), a key inflammatory marker, is the precursor of AA and is sharply elevated during inflammation [8]. Calcium overload in cells is a very important factor in the pathological process of amyloidosis [9,10]. Strong evidence indicates that soluble amyloid causes an imbalance in calcium homeostasis by promoting an uncontrolled rise in cytoplasmic Ca^2+^ levels [11,12]. The imbalance of calcium homeostasis not only promotes the accumulation and deposition of amyloid proteins, but also directly activates inflammatory signaling pathways [13]. Excessive expression of inflammatory factors upregulates Phosphodiesterase 5A (PDE5A) levels, which consequently suppresses the activation of the cyclic guanosine monophosphate/Protein kinase G (cGMP/PKG) axis [14,15]. The cGMP/PKG signaling pathway plays a key role in regulating inflammation and maintaining calcium homeostasis. PKG activation reduces the intracellular Ca^2+^ concentration, thereby downregulating the expression of pro-inflammatory factors IL-6 and TNF-α, and ultimately suppressing the progression of inflammation [16,17]. However, strategies to ameliorate HA by the cGMP/PKG signaling pathway remain insufficiently explored.

Current therapeutic strategies for AA amyloidosis mainly focus on treating the underlying etiology, enhancing anti-inflammatory effects, reducing amyloid precursor production [18], inhibiting amyloid fibril synthesis [19], decreasing tissue deposition, and promoting fibril dissolution [20]. Currently, therapeutic approaches for amyloidosis still face several challenges, including potential immunogenicity, high treatment costs, and the need for further long-term efficacy evidence from clinical studies [21].

In traditional Chinese medicine (TCM), amyloidosis is viewed as a pathological state arising from visceral dysfunction and impaired movement of qi, blood, and body fluids [22,23]. Paeoniae Radix Alba Carbonisata (PRAC) is a carbonized of decoction piece obtained by stir-baking the peeled root of *Paeonia lactiflora* Pall. over strong heat until the surface becomes brownish-black and the interior dark brown. It is commonly used as a liver-protective agent in TCM, attributed to its actions of cooling blood to stop bleeding and calming liver yang [24]. PRAC scavenged reactive oxygen species, inhibited lipid peroxidation, and ameliorated inflammatory responses in liver cells [25]. Furthermore, Paeoniflorin from peony root regulated abnormal neurofibrillary tangles of Tau protein and excessive accumulation of β-amyloid protein to reduce inflammation, oxidative stress, and neuron apoptosis in Alzheimer’s disease (AD) animal models [26,27]. However, the roles of PRAC in HA are still unclear.

Our preliminary research found PRAC extract (PRAC-E) enhanced NCTC1469 cell viability, reduced inflammation, and alleviated the increase in cytoplasmic Ca^2+^ concentration induced by amyloid fibers. Based on these findings, we hypothesized that restoring calcium homeostasis and regulating the inflammatory response may be a potential strategy for PRAC-E intervention in HA.

In this paper, we explored and verified the intervention mechanisms of PRAC-E on amyloid-induced hepatocyte injury in vivo and in vitro. Furthermore, the UHPLC-Q-Exactive Orbitrap MS technique was used to characterize the chemical components in PRAC-E, and the core components were quantified by UHPLC-QTRAP-MS/MS.

## 2. Results

### 2.1. PRAC-E Against Protein Amyloid-Induced Liver Injury

**Figure 1:**
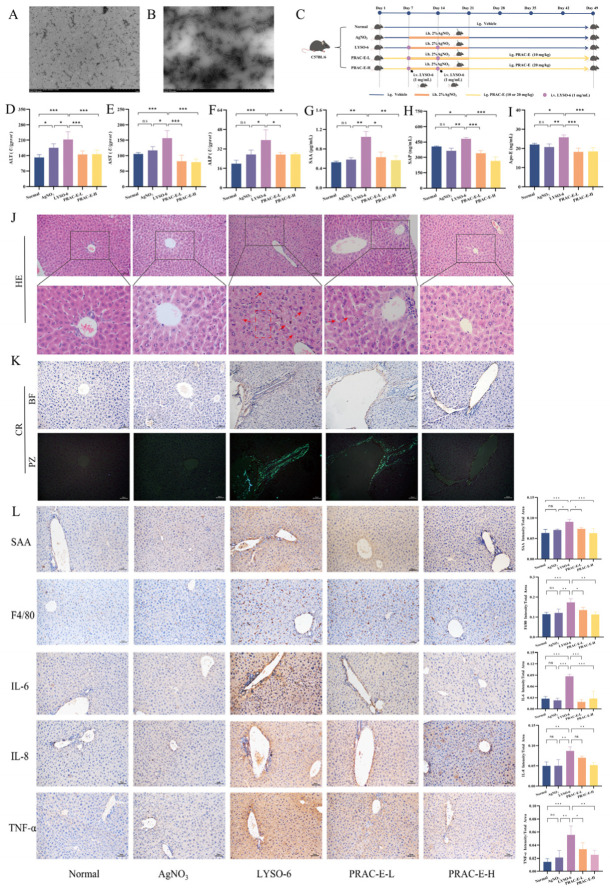
PRAC-E alleviated liver injury induced by LYSO-6. (**A**,**B**) Transmission electron microscopy (TEM) images of normal Hen egg-white lysozyme (LYSO) and LYSO-6. (**C**) Flow chart of animal treatment. (**D**–**F**) The levels of serum ALT, AST, and ALP. (**G**–**I**) The levels of SAA (**G**), SAP (**H**), and Apo-E (**I**) in liver tissue. (**J**) H&E staining. (**K**) CR staining (PZ: polarization, BF: brightfield). (**L**) IHC staining and semi-quantitative for SAA, F4/80, IL-6, IL-8, and TNF-α expression in liver sections (200×). Data are presented as the mean ± standard deviation (SD) (*n* = 6). * *p* < 0.05, ** *p* < 0.01, *** *p* < 0.001 vs. LYSO-6; ns, no significance.

We successfully prepared amyloid lysozyme (LYSO-6) (Figure 1A,B and Figure S3) to construct a liver injury model stimulated by amyloid fibrils in vivo. The AgNO_3_ group and the Normal group did not show significant statistical differences in hematological parameters and tissue morphology. In contrast, LYSO-6 induction markedly elevated Alanine aminotransferase (ALT), Aspartate aminotransferase (AST), and Alkaline phosphatase (ALP) levels (*p* < 0.001) and induced liver injury, all of which were significantly alleviated by oral administration of PRAC-E (Figure 1D–F; *p* < 0.05). Immunohistochemical analysis demonstrated that the expression of inflammatory markers (SAA, F4/80, IL-6, IL-8, and TNF-α) was significantly upregulated (*p* < 0.01) following LYSO-6 induction, but markedly downregulated after PRAC-E intervention (Figure 1L; *p* < 0.05). Similarly, levels of the amyloid-associated proteins SAA, Serum amyloid P component (SAP), and Apolipoprotein E (Apo-E) in liver tissue were elevated (*p* < 0.05) after LYSO-6 induction. These increases were inhibited by PRAC-E (Figure 1G–I; *p* < 0.05). In the LYAO-6 group, evident cellular damage was observed. Hepatocytes exhibited enlarged morphology, cytoplasm vacuolation, and nuclei that had shrunk to varying degrees (indicated by the dashed line in Figure 1J). Furthermore, homogeneous eosinophilic deposits were noted within the hepatic sinusoids (indicated by the arrow). PRAC-E improved these histological changes. Congo red (CR) staining showed no amyloid deposition in the blank group and AgNO_3_ group. In the LYSO-6 group, a large amount of amyloid deposition was found around the vessel wall under an optical microscope, and a homogenized orange-red substance was seen at the deposition site; meanwhile, apple green birefringent light was shown under a polarized light microscope (Figure 1K). Oral administration of PRAC-E successfully reduced amyloid deposition and associated histopathological damage.

### 2.2. Transcriptomic Analysis and Ca^2+^-ATPase Activity of the PRAC-E Intervention on HA

**Figure 2:**
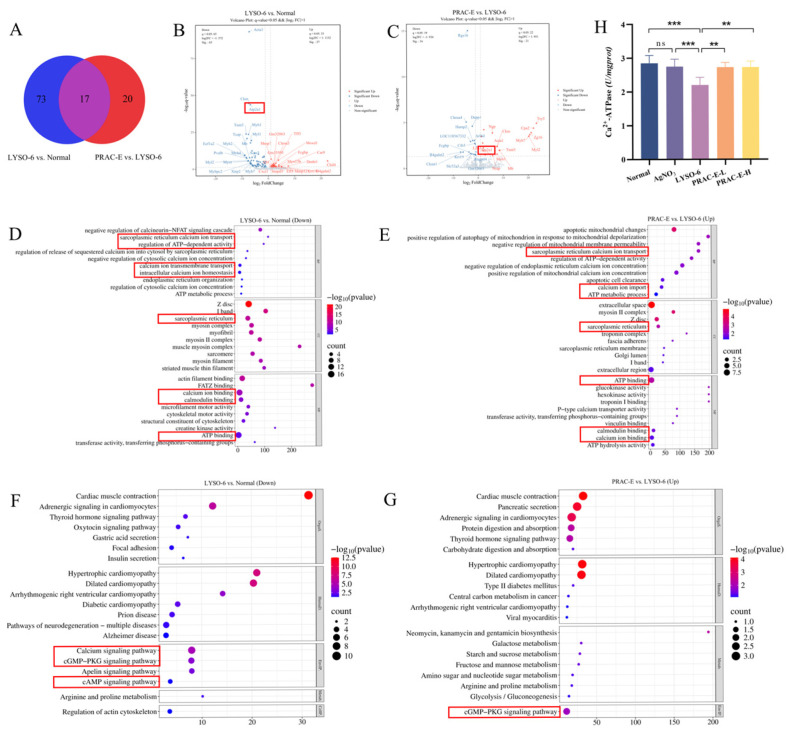
Transcriptomic results of PRAC-E ameliorating HA. (**A**) Venn diagram of the DEGs from PRAC-E vs. LYSO-6 and LYSO-6 vs. Normal. (**B**) LYSO-6 vs. Normal DEGs volcano plot. (**C**) PRAC-E vs. LYSO-6 DEGs volcano plot. (**D**) Functional enrichment analysis of downregulated DEGs in LYSO-6 vs. Normal. (**E**) Function enrichment analysis of upregulated DEGs in PRAC-E vs. LYSO-6. (**F**) Pathway enrichment analysis of the downregulated DEGs in LYSO-6 vs. Normal. (**G**) Pathway enrichment analysis of upregulated DEGs in PRAC-E vs. LYSO-6. (**H**) Ca^2+^-ATPase activity in liver tissue (*n* = 6). Data are presented as the mean ± standard deviation (SD) (*n* = 6). ** *p* < 0.01, *** *p* < 0.001 vs. LYSO-6.

Venn diagram analysis identified 90 differentially expressed genes (DEGs) between the Normal and LYSO-6 groups and 37 DEGs between the LYSO-6 and PRAC-E-treated groups (Figure 2A). Volcano plot analysis highlighted ATP2A1 as a key gene that was significantly downregulated in the LYSO-6 group and upregulated after PRAC-E treatment (Figure 2B,C). To elucidate the associated biological functions, we performed GO and KEGG enrichment analyses on the top 20 significant pathways (Figure 2D–G). The LYSO-6 group exhibited downregulation in processes including calcium ion transport and ATP metabolism, which were reversed by PRAC-E treatment. The LYSO-6 group significantly downregulated key signaling pathways, including the calcium signaling pathway, cAMP signaling pathway, and cGMP/PKG signaling pathway. Notably, PRAC-E treatment restored the expression of genes within the cGMP/PKG signaling pathway. We systematically validated the Ca^2+^-ATPase activity in liver tissue, and the results showed that the Ca^2+^-ATPase activity in the LYSO-6 group was significantly lower (*p* < 0.001) than in the Normal group but was significantly restored by PRAC-E treatment (Figure 2H; *p* < 0.01).

### 2.3. PRAC-E Reduces Ca^2+^ Overload by cGMP/PKG/ATP2A1 Signaling Axis

**Figure 3:**
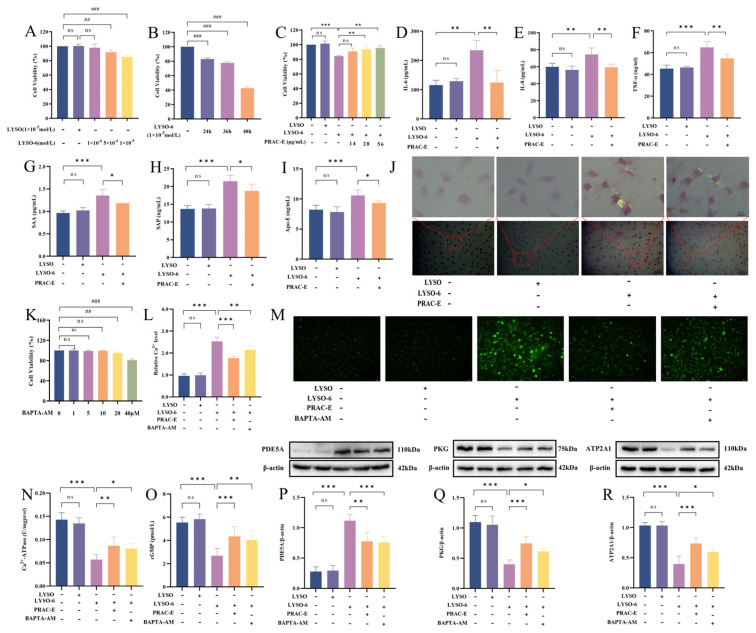
PRAC-E modulates calcium homeostasis via the cGMP/PKG/ATP2A1 signaling axis, thereby alleviating inflammation and amyloid deposition. (**A**,**B**) The dose-dependent (**A**) and time-dependent (**B**) effects of LYSO-6 on NCTC1469 cells. (**C**) Screening of the effective concentration of PRAC-E on LYSO-6-stimulated cells. (**D**–**F**) The levels of IL-6, TNF-α, and IL-8 in the cell culture supernatant. (**G**–**I**) The levels of SAA, SAP (**G**), and APO-E (**H**) in NCTC1469 cells. (**J**) CR staining of NCTC1469 cells. (**K**) The dose-dependent effects of BAPTA-AM on NCTC1469 cells. (**L**,**M**) Representative Fluo-4 AM fluorescence staining and semi-quantification of NCTC1469 cells. (**N**) Ca^2+^-ATPase activity in NCTC1469 cells. (**O**) Intracellular cGMP levels in NCTC1469 cells were measured by ELISA. (**P**–**R**) Western blotting and semi-quantitative analysis of PDE5A, PKG, and ATP2A1 in NCTC1469 cells. Data are presented as the mean ± SD (*n* = 6). ^#^ *p* < 0.05, ^##^ *p* < 0.01, ^###^ *p* < 0.001 vs. solvent control group; * *p* < 0.05, ** *p* < 0.01 and *** *p* < 0.001 vs. LYSO-6; ns, no significance.

There was no significant difference in the cell viability and morphology between the LYSO treatment group and the solvent control group. Compared with the solvent control group, 1 × 10^−5^ mol/L LYSO-6 significantly decreased the viability of NCTC1469 cells after 24 h (Figure 3A,B). The protective effect of PRAC-E against LYSO-6-induced cytotoxicity was dose-dependent, with cell viability increasing progressively at higher concentrations. At a concentration of 28 μg/mL, PRAC-E restored cell viability to 93.68 ± 4.09% (*p* < 0.01), showing a significant protective effect. Therefore, 28 μg/mL of PRAC-E was selected for subsequent experiments (Figure 3C). The levels of IL-6, IL-8, and TNF-α in the LYSO-6 group were significantly increased (*p* < 0.01), and PRAC-E treatment reduced the levels of inflammatory factors (Figure 3D–F; *p* < 0.01). The levels of SAA, SAP, and Apo-E were significantly increased (*p* < 0.001) by LYSO-6 treatment, which were reduced by PRAC-E (Figure 3G–I; *p* < 0.05). In the LYSO-6 group, CR staining of hepatocytes showed that the amyloid protein was deposited between cells. After PRAC-E intervention, amyloid fiber deposition was alleviated (Figure 3J).

BAPTA-AM, a well-known membrane-permeable Ca^2+^ chelator, was used to prevent cell injury by reducing intracellular calcium overload [28]. We assessed cell viability using the CCK-8 assay and conducted subsequent experiments within the safe concentration range of BAPTA-AM (Figure 3K; *p* > 0.05). Measurement of intracellular calcium ions showed that LYSO-6 significantly increased (*p* < 0.001) the calcium ion level in the cells, and this was significantly reduced by PRAC-E (Figure 3L,M; *p* < 0.001). The Ca^2+^-ATPase activity in hepatocytes showed that after LYSO-6 stimulation, the Ca^2+^-ATPase activity was significantly reduced (*p* < 0.001), but PRAC-E intervention significantly restored its activity (Figure 3N; *p* < 0.01). We investigated the protein expression of cGMP/PKG/ATP2A1 signaling axis in NCTC1469 cells (Figure 3O–R and Figure S1). LYSO-6 stimulation notably upregulated the expression of PDE5A and downregulated (*p* < 0.001) the expression of cGMP, PKG, and ATP2A1, compared to the solvent control group. PRAC-E and BAPTA-AM effectively reversed the altered expression of these proteins (*p* < 0.05).

### 2.4. Core Component Mining and Analysis from PRAC-E Intervention in HA

**Figure 4:**
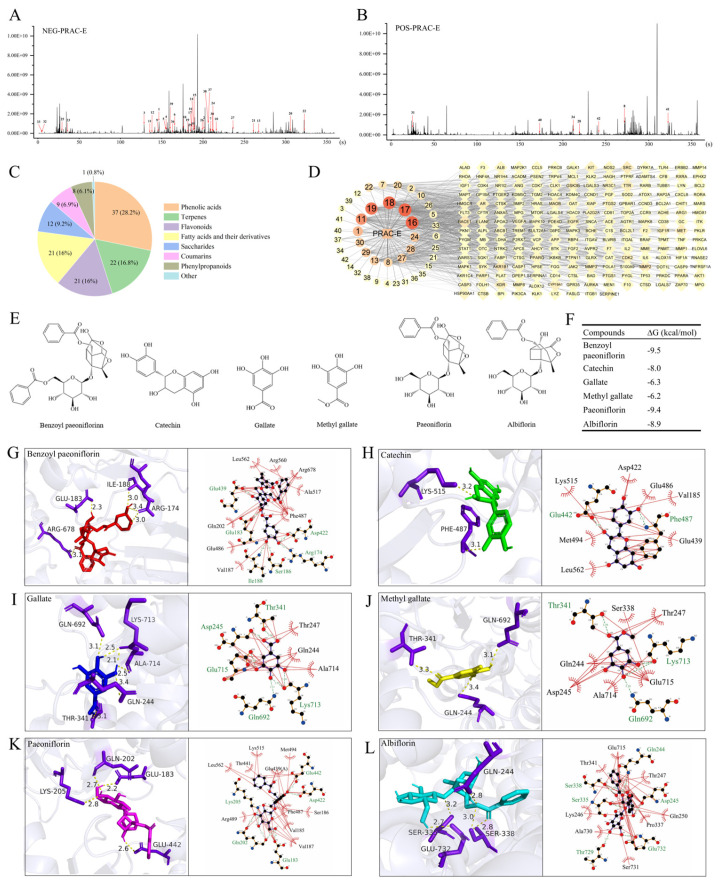
Characterization of components in PRAC-E and analysis of core components. (**A**,**B**) Total ion flow diagram of PRAC-E under negative (**A**) and positive (**B**) ion mode. Number 1 to 42: The active ingredients are detailed in Table 1. (**C**) The types of compounds in the PRAC-E. (**D**) The TCM–component–target network. (**E**) Six core component structure diagrams. (**F**) Molecular docking binding energy. (**G**) Benzoyl paeoniflorin-ATP2A1. (**H**) Catechin-ATP2A1. (**I**) Gallate-ATP2A1. (**J**) Methylgallate-ATP2A1. (**K**) Paeoniflorin-ATP2A1. (**L**) Albiflorin-ATP2A1. Note: In Figure 4G–L, the green font indicates non-ligand residue names, and the black font indicates hydrophobic residue names.

A total of 131 components of PRAC-E were identified, including 28 identified in positive ion mode and 103 identified in negative ion mode (Figure 4A,B). As illustrated in Figure 4C, approximately 61% of the components were phenolic acids, terpenoids, and flavonoids. The names of each component, retention time, molecular formula, component classification, and other details were listed in Table S1. An interaction network of TCM components and targets was constructed to intuitively visualize the potential key components and targets of PRAC-E in the intervention of hepatic amyloidosis (Figure 4D). According to the ranking of degree values (Table 1), six core components (SCCs) were identified as Benzoyl paeoniflorin, Catechin, Gallate, Methyl gallate, Paeoniflorin, and Albiflorin (Figure 4E and Figure S2; Tables S2–S5). The binding energies between the SCCs and ATP2A1 were all lower than −6.2 kcal/mol, indicating the formation of relatively stable complexes (Figure 4F–L). The quantitative analysis results showed that Gallate (16.96 mg/g), as the representative component of phenolic acid, had a higher content than that of Paeoniflorin (14.27 mg/g), the representative component of terpenoids (Table 2).

## 3. Discussion

Currently, stimulants used in amyloidosis research are primarily derived from amyloid-enhancing factors (AEFs) extracted from animal tissues affected by amyloidosis [29]. These factors act as nuclei for fibril formation, promoting the formation and tissue deposition of amyloid fibrils through cross-”seeding” effects in vivo [30]. However, AEFs are characterized by complex and heterogeneous composition, including amyloid fibrils [31], SAP [32], heparan sulfate [33], and other components. This complexity makes it difficult to precisely distinguish the independent roles of each component in cross-nucleation. Their activity is influenced by extraction methods, animal strains, and disease stages, resulting in poor experimental reproducibility and high costs. Furthermore, non-specific proteins present in AEFs may interfere with the accurate analysis of amyloid fibril deposition mechanisms, limiting the screening and validation of key pathogenic targets [34]. We utilized structurally well-defined and readily available hen egg-white lysozyme to prepare low-cost exogenous amyloid seeds and successfully established both in vitro and in vivo models of amyloidosis [35]. These models exhibit similar pathological features to those reported in clinical amyloidosis studies, characterized by enhanced inflammatory responses and amyloid deposition.

Calcium overload is related to the progression of amyloid protein deposition [36,37]. ATP2A1 (SERCA1) is an endoplasmic reticulum calcium pump that maintains calcium equilibrium [38]. Increased ATP2A1 expression contributes to the restoration of calcium homeostasis and the alleviation of pathological damage [38]. Calcium homeostasis is crucial for ensuring normal protein synthesis, folding, and secretion, and mitigates the damage to cellular functions [39]. As an important second messenger, the maintenance of calcium homeostasis blocks abnormal inflammatory signaling and reduces the release of inflammatory factors, thereby significantly inhibiting inflammatory responses [40]. Studies have shown that calcium overload leads to increased mitochondrial reactive oxygen species expression, subsequently activating the NLRP3 inflammasome, and promoting the release of inflammatory factors such as TNF-α [41,42]. This cascade ultimately upregulates the expression of PDE5A, a cGMP-specific phosphodiesterase, resulting in a significant reduction in intracellular cGMP levels. Furthermore, previous studies have demonstrated that upregulation of both PDE5A expression and its enzymatic activity significantly reduces cGMP levels [43,44]. This reduction in cGMP suppresses the activation of the cGMP/PKG pathway [45]. The inhibition of this pathway may further aggravate the dysfunction of hepatocytes [46].

PRAC has been documented to possess the TCM efficacy of “protecting the liver” [25]. However, modern research on its hepatoprotective effects remains limited. We found PRAC-E upregulated ATP2A1 expression and enhanced Ca^2+^-ATPase activity to reduce the cellular inflammatory response. This effect contributed to reducing PDE5A levels and activating the cGMP/PKG signaling pathway, which helped alleviate calcium overload and maintain calcium homeostasis. Gallate and Paeoniflorin were demonstrated to possess broad-spectrum anti-inflammatory [47,48], antioxidant and immunomodulatory effects [49,50]. In addition, studies have shown that the above components can inhibit the reduction in β-folding content in an in vitro chemical environment and simultaneously increase the proportion of α-helix [51,52], which is beneficial to prevent the formation and abnormal accumulation of amyloid fibrils. We also further demonstrated through multispectral analysis that both PRAC-E and its core components significantly inhibited amyloid fibril formation in vitro (Figures S3–S5). These findings further highlight the credibility of SCCs we predicted in PRAC-E. SCCs align with traditional uses while also offering a solid scientific foundation for their hepatoprotective effects.

Systemic amyloidosis often involves multiple organs and exhibits complex pathogenic mechanisms [53]. TCM is characterized by the core strengths of multi-component synergy and holistic multi-target regulation, which may be well-suited for targeting the complex pathological network involved in the prevention and treatment of systemic amyloidosis. Further research is needed to subsequently conduct research on the holistic regulatory effects of multi-components and elucidate their synergistic mechanisms in targeting amyloid fibrillation guided by the above insights.

## 4. Materials and Methods

### 4.1. Chemicals and Reagents

Hen egg-white lysozyme (LYSO) was purchased from Dalian Meilun Biotechnology Co., Ltd. (Dalian, China). PRAC was procured from Anhui Jingwan Pharmacy Chain Co., Ltd. (Bozhou, China). Dulbecco’s modified Eagle medium (DMEM) and fetal bovine serum (FBS) were purchased from Gibco (Carlsbad, CA, USA). Hematoxylin and eosin dye solution was from Zhuhai Baso Biotechnology Co., Ltd. (Zhuhai, China). The CCK-8 kit was purchased from GLPBIO (Montclair, CA, USA). The Fluo-4 AM fluorescence calcium ion detection kit from Beyotime Biotechnology (Shanghai, China). The Universal kit (mouse/rabbit polymer detection system, PV-6000) from Beijing Zhongshan Golden Bridge Biotechnology Co., Ltd. (Beijing, China). Congo red was obtained from Damas-beta (Shanghai, China). Mouse SAA, SAP, Apo-E, and IL-8 ELISA kits were purchased from Shanghai ZCIBIO Technology Co., Ltd. (Shanghai, China). The BCA protein quantitative kit, as well as mouse IL-6 and TNF-α ELISA kits, were obtained from ABclonal Biotechnology Co., Ltd. (Wuhan, China). SAA and F4/80 antibody were also sourced from ABclonal. The cGMP ELISA Kit was sourced from Elabscience Biotechnology Co., Ltd. (Wuhan, China). ALT, AST, ALP, and ultramicro Ca^2+^-ATPase detection kits were obtained from Nanjing Jiancheng Bioengineering Institute (Nanjing, China). Antibodies against IL-6 (Cat# WL02841), TNF-α (Cat# WL01581), and IL-8 (Cat# WL03074) were purchased from Shenyang Wanlei Life Sciences Co., Ltd. (Shenyang, China). Rabbit antibodies against β-actin (Cat# 20536-1-AP), PDE5A (Cat# 22624-1-AP), and ATP2A1 (Cat# 22361-1-AP) were obtained from Proteintech Group, Inc. (Wuhan, China). The PKG antibody (Cat# bs-6705R) was purchased from Bioss (Beijing, China), and NcmECL Ultra was purchased from NCM Biotech (Suzhou, China).

### 4.2. Preparation of Solvents and Test Samples

Solution pH = 2: A solution with pH 2.0 was prepared by slowly adding concentrated hydrochloric acid to ultrapure water under pH meter monitoring.

LYSO solution: Accurately weigh an appropriate amount of LYSO powder, dissolve it in a solution with pH = 2, and the final concentration is 1 × 10^−3^ mol/L. After completely dissolving, filter twice using a 0.45 μm filter membrane and dilute as needed before use.

Preparation of lysozyme amyloid fibrils (LYSO-6): The LYSO solution was placed in a constant temperature oscillator (CYLY Instrument, TS-2112B, Changzhou, China) at 65 °C and 50 rpm for 6 days. Filter with a 0.22 μm filter membrane and dilute as needed before use.

Authentication of commercially procured PRAC: According to the processing standard for PRAC [54], the authenticity of the medicinal material was rigorously verified by Professor Lijian Cui at Hebei University of Chinese Medicine.

Preparation of PRAC-E: PRAC was pulverized and sieved through an 80-mesh sieve. Weigh 30.0 g powder into a 2000 mL Bunsen flask, add 1000 mL of 50% ethanol for cold maceration for 24 h, followed by ultrasonication (300 W, 45 kHz) for 30 min [55]. Cool and filter, and then dry in a freeze dryer (Wuxi Voshin Instruments Co., Ltd., FD-1A-50, Wuxi, China) to obtain PRAC-E lyophilized powder.

Preparation of qualitative and quantitative PRAC-E test solutions: PRAC-E lyophilized powder (1.00 mg) was dissolved in 2 mL of solvent (methanol:acetonitrile:water = 2:2:1, *v*/*v*/*v*). The resulting solution was filtered through a 0.45 μm filter membrane before use.

### 4.3. Transmission Electron Microscopy

A total of 5 μL of LYSO or LYSO-6 sample (1 × 10^−4^ mol/L) was transferred to a copper grid, followed by the addition of 10 μL of 1% phosphotungstic acid. The samples were observed using a TEM (HITACHI, 7800, Tokyo, Japan) under an acceleration voltage of 80 kV.

### 4.4. Establishment and Treatment of the Amyloidosis Mouse Model

An HA model was established in Male C57BL/6 mice (6–8 weeks, weighing 19–21 g) referencing Vahdat’s literature [56]. The mice were randomly assigned to 5 groups (*n* = 6 per group): a vehicle group, a AgNO_3_ group, a LYSO-6 group, and two PRAC-E groups (low and high doses) (Figure 1C). All animals were acclimatized for one week in a specific pathogen-free environment 12 h light/12 h dark cycle at a room temperature of 25 °C ± 1 °C and humidity of 60% ± 5% before the onset of any experiments. Then, PRAC-E dissolved in physiological saline containing 0.1% DMSO was administered orally to mice once daily at doses of 60 or 120 mg/kg in the PRAC-E groups for 7 weeks. The mice in other groups received an equal volume of vehicle. From the 8th day, the mice in the AgNO_3_ group, LYSO-6 group, and PRAC-E groups received daily subcutaneous injections of 2% *w*/*v* AgNO_3_ (0.2 g/kg) on the dorsal skin for two weeks, additionally, the mice in the LYSO-6 group and PRAC-E groups were administered LYSO-6 (5 mg/kg) via tail vein injection once per week for two weeks. On the 49th day, mice were euthanized, and liver and blood samples were collected.

### 4.5. Cell Culture

Mouse NCTC1469 liver cell line was purchased from Sebikon Biotechnology Co., Ltd. (Shanghai, China). Cells were cultured in DMEM (containing 10% FBS, 100 U/mL penicillin, and 100 U/mL streptomycin) at 37 °C and 5% CO_2_. The cells were passaged upon reaching 90% confluence.

### 4.6. Establishment and Treatment of the Amyloid-Induced Cell Model

NCTC1469 cells (1 × 10^4^ cells/well) were seeded in 96-well plates and cultured in an incubator containing 3% FBS, 100 U/mL penicillin/streptomycin, and 5% CO_2_ at 37 °C for 24 h. Cells were divided into the following groups: Normal group, LYSO group, LYSO-6 group, and PRAC-E groups. The cells in PRAC-E groups were pretreated with PRAC-E (14, 28, and 56 μg/mL) for 2 h, followed by the addition of LYSO-6 (1 × 10^−5^ mol/L) in the LYSO-6 group and PRAC-E groups for 24 h. The other two groups of cells were treated with an equivalent volume of vehicle or LYSO (1 × 10^−5^ mol/L).

### 4.7. Cell Viability Assay

Cell viability was assessed by the CCK-8 assay according to the CCK-8 manufacturer’s instructions (Beyotime, C0038, Jiangsu, China). Cells were seeded onto 96-well plates in sextuplicate. CCK-8 reagent was added to the cells and incubated in the incubator for 2 h, after which optical density (OD) values were recorded at 450 nm using a microplate reader.

### 4.8. Biochemical Parameter Detection

Equal weights of liver tissue were homogenized in physiological saline at a ratio of 1:9 (*w*/*v*) with protease inhibitors at 1:100 (*v*/*v*). The tissues were pulverized by a cryogenic grinder and centrifuged at 4000 rpm for 10 min at 4 °C. The supernatants were diluted 50-fold for protein quantification using the BCA protein assay kit. Commercial kits were used to evaluate the levels of SAA, SAP, Apo-E, cGMP, and intracellular Ca^2+^ concentration, as well as the activity of Ca^2+^-ATPase.

Serum samples were used to measure ALT, AST, ALP, IL-6, IL-8, and TNF-α levels.

The extracellular secreted proteins were directly measured from the above supernatant.

NCTC1469 cells were detached from culture dishes to centrifuge tubes for cell samples. Sonication lysed cells, followed by the centrifugation of the cell suspension (4000 rpm, 5 min).

### 4.9. Histopathological Examination of the Liver Tissues

Liver tissues from C57BL/6 mice were fixed in 10% buffered formalin, processed through a graded alcohol series, cleared in xylene, and embedded in paraffin. The embedded tissues were sectioned into 5 μm-thick slices and stained with H&E or CR [57]. Images were acquired using a polarizing microscope (SOPTOP, CX40P, Ningbo, China).

### 4.10. Immunohistochemistry Analysis

Following deparaffinization with xylene and hydration through a graded ethanol series, antigen retrieval was performed by heating the sections in citrate buffer for 15 min. Endogenous peroxidase activity was then blocked by incubation with 3% hydrogen peroxide. The sections were blocked with 5% goat serum for 1 h to minimize nonspecific binding, followed by overnight incubation at 4 °C with primary antibodies against F4/80, SAA, IL-6, TNF-α, or IL-8 (1:200). After washing with PBS, the sections were incubated with horseradish peroxidase-conjugated secondary antibodies for 1 h at room temperature. Color development was achieved using a 3,3′-diaminobenzidine (DAB) substrate, followed by counterstaining with hematoxylin. Finally, the sections were dehydrated, cleared, and imaged under a light microscope (Nikon, Tokyo, Japan). Image analysis was performed using Image-Pro Plus 6.0 software (Media Cybernetics, Rockville, MD, USA).

### 4.11. RNA Isolation and Library Preparation

Total RNA was extracted using the TRIzol reagent (Invitrogen, Carlsbad, CA, USA) according to the manufacturer’s protocol. RNA purity and quantification were evaluated using the NanoDrop 2000 spectrophotometer (Thermo Fisher Scientific, Wilmington, MA, USA). RNA integrity was assessed using the Agilent 2100 Bioanalyzer (Agilent Technologies, Santa Clara, CA, USA). Then the libraries were constructed using the VAHTS Universal V6 RNA-seq Library Prep Kit according to the manufacturer’s instructions.

### 4.12. RNA Sequencing and Differentially Expressed Genes Analysis

The libraries were sequenced on an Illumina Novaseq 6000 (Illumina, Inc., San Diego, CA, USA) platform and 150 bp paired-end reads were generated. Raw reads of fastq format were first processed using fastp [58], and the low-quality reads were removed to obtain the clean reads. Then, approximately 18 million clean reads for each sample were retained for subsequent analyses. The clean reads were mapped to the reference genome using HISAT2 [59]. FPKM [60] of each gene was calculated, and the read counts of each gene were obtained by HTSeq-count [61]. Differential expression analysis was performed using the DESeq2 [62]. Q value < 0.05 and foldchange > 2 or foldchange < 0.5 were set as the threshold for significant DEGs. The radar map of the top 30 genes was drawn to show the expression of upregulated or downregulated DEGs using R package (v3.5.1)“ggplot2”. Based on the hypergeometric distribution, GO, and KEGG pathway enrichment analysis of DEGs were performed to screen the significantly enriched term using R (v 3.2.0), respectively.

### 4.13. Intracellular Ca^2+^ Ions Examination

The Fluo-4 AM stock solution was diluted in a serum-free culture medium to a working concentration of 2 µM. The diluted solution was added to the cells and incubated in a CO_2_ incubator in the dark for 30 min. After incubation, the staining solution was removed, and the cells were washed twice with pre-cooled PBS to remove any probe that had not entered the cells. Fluorescence intensity was measured by a fluorescence microscope (Olympus Corporation, Hachioji-shi, Tokyo, Japan).

### 4.14. Western Blotting

Proteins were extracted from cells using RIPA lysis buffer supplemented with protease and phosphatase inhibitors. Protein concentration was determined with a BCA assay kit. Protein samples were separated using 10% sodium dodecyl sulfate-polyacrylamide gel electrophoresis and transferred onto a polyvinylidene fluoride membrane. Subsequently, the membrane was blocked with 5% non-fat milk and incubated overnight at 4 °C with the following primary antibodies: β-actin (1:40,000), PDE5A (1:4000), PKG (1:500), and ATP2A1 (1:5000). Afterward, the membrane was incubated with the corresponding horseradish peroxidase (HRP)-conjugated secondary antibodies (1:4000) for 2 h at room temperature. Protein bands were visualized using an NcmECL Ultra chemiluminescence substrate (New Cell and Molecular Biotech Co., Ltd., Suzhou, China). The signal intensities of the bands were semi-quantified by densitometry using Image Pro Plus 6.0 (Media Cybernetics, Rockville, MD, USA), and the protein expression levels were normalized to the expression level of β-actin.

### 4.15. UHPLC-Q-Exactive Orbitrap MS Analysis

Chromatographic separation was performed on a Kinetex C18 column (2.1 mm × 50 mm, 2.6 μm). The mobile phase consisted of 0.1% formic acid in water (A) and acetonitrile (B), and the gradient elution program was as follows: 0–0.5 min, 1% B; 0.5–4.0 min, 1–99% B; 4.5–4.55 min, 99–1% B; 4.55–6 min, 1% B. The flow rate was set at 0.3 mL/min, and the injection volume was 2 μL.

Mass spectrometry was performed using Q-Exactive Orbitrap MS/MS (Thermo Fisher Scientific, USA). The ion source was heated electrospray (HESI, 350 °C), and the samples were acquired in data-dependent acquisition mode, with a primary scanning resolution of 60,000, a secondary scanning resolution of 15,000, and a scanning range of *m*/*z* 50–1500; the positive and negative ion detection modes were used, with a transfer capillary temperature of 320 °C; the sheath gas was 50 psi, and the flow rate of auxiliary gas was 15 psi spray voltage of 3.8 kV (positive) or −3.4 kV (negative).

The raw data were converted to mzXML format using ProteoWizard software 3.0.18 (ProteoWizard Software Foundation, Palo Alto, CA, USA). The converted files were then matched against the BiotreeDB (v3.0) (https://www.biotree.cn/en/ (accessed on 2 August 2025)) and HMDB (https://hmdb.ca/ (accessed on 2 August 2025)) databases for component identification.

### 4.16. UHPLC-QTRAP-MS/MS Analysis

Chromatographic separation was performed on an Agilent Poroshell 120 EC-C18 column (2.1 × 100 mm, 2.7 μm) maintained at 35 °C. The mobile phase consisted of ultrapure water (A) and acetonitrile (B). A gradient elution program was applied as follows: 0–2 min, 5–13% B; 2–5 min, 13–19% B; 5–10 min, 19–33% B; 10–12 min, 33–55% B; 12–15 min, 55–100% B; 15–18 min, 100–5% B. The flow rate was 0.3 mL/min, and the injection volume was 5 μL.

Mass spectrometric detection was conducted using an ESI source in negative ion mode with multiple reaction monitoring (MRM). The ion source parameters were set as follows: ion spray voltage, −4500 V; source temperature, 650 °C. Data acquisition was performed using the Analyst 1.5.2 software (Sciex, Framingham, MA, USA), and quantitative analysis was carried out with MultiQuant 3.0.2 software (Sciex, Framingham, MA, USA).

### 4.17. Network Pharmacology

The targets of PRAC-E components were retrieved from the online PharmMapper (http://www.lilab-ecust.cn/pharmmapper/ (accessed on 8 August 2025)) and SWISS Target Prediction database (http://www.swisstargetprediction.ch/ (accessed on 8 August 2025)). Predictions with a probability score > 0.06 were retained and standardized using the UniProt database (https://www.uniprot.org/ (accessed on 8 August 2025)). Disease-related targets for HA were identified by searching the OMIM (https://omim.org/ (accessed on 9 August 2025)) and GeneCards (https://www.genecards.org/ (accessed on 9 August 2025)) databases with the keywords “hepatic amyloidosis” and “amyloidosis of the liver”. The results were filtered for *Homo sapiens* and duplicates were removed. Overlapping targets between the PRAC-E components and HA were considered candidate targets. These were imported into Cytoscape 3.9.1 to construct a “Component-Target-Pathway” network. The network was analyzed using the “Network Analyzer” tool to visualize complex relationships, and core components were identified based on a degree value ≥ 10.

### 4.18. Molecular Docking

The bindings of ATP2A1 with the SCCs of PRAC-E were simulated using AutoDock Tools-1.5.6 (Scripps Institute, La Jolla, CA, USA). The three-dimensional (3D) structure of ATP2A1 (PDB ID: 3ARF) was obtained from the Protein Data Bank (https://www.rcsb.org/ (accessed on 13 August 2025)). The 3D structures of the SCCs of PRAC-E were downloaded from the PubChem database (https://pubchem.ncbi.nlm.nih.gov/ (accessed on 13 August 2025)). Water molecules were removed from the ATP2A1 structure, Gasteiger partial charges were assigned, and non-polar hydrogen atoms were merged. A grid box with dimensions of 70 × 70 × 126 Å was created, centered at coordinates (x = 41.2, y = −2.9, z = 108.9). The lowest-energy docked conformation was selected for further analysis and visualization using PyMOL 2.5.0 (DeLano Scientific LLC, Palo Alto, CA, USA) and LigPlot^+^ v.2.3 software (EMBL-EBI, Hinxton, Cambridgeshire, UK).

### 4.19. Statistical Analysis

All experiments were performed at least 3 times. Data are expressed as means ± SD. The data were evaluated using SPSS 19.0 (International Business Machines Corporation, Armonk, NY, USA). For pairwise comparisons of the means of multiple samples, one-way ANOVA was used, and a value of *p* < 0.05 was considered statistically significant.

## 5. Conclusions

**Figure 5:**
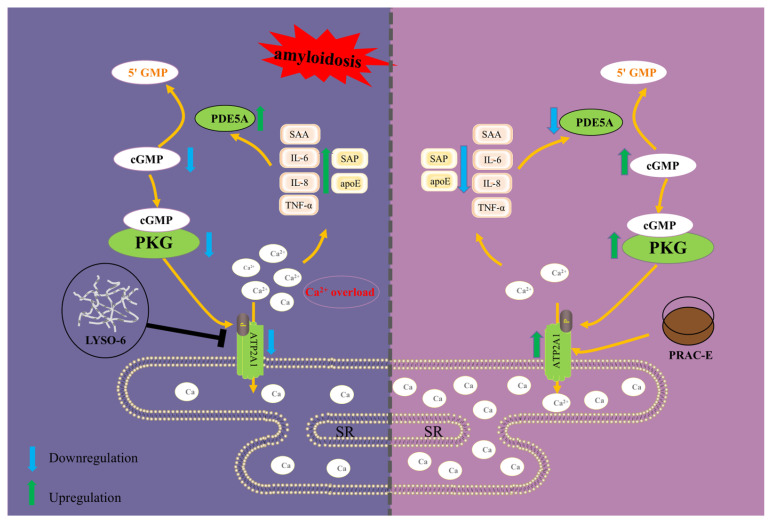
The possible mechanism by which PRAC-E intervention in amyloidosis liver injury.

This study systematically investigated the mechanism of PRAC-E in amyloidosis-related liver injury. The results showed that LYSO-6 induced secondary amyloidosis in the liver through the “seed” effect, accelerating the progression of amyloid-related liver damage. PRAC-E regulated calcium homeostasis to ameliorate HA (Figure 5). Through qualitative, quantitative, and network pharmacological analyses of PRAC-E, potential SCCs for alleviating HA were identified. Molecular docking revealed that all SCCs bound strongly to ATP2A1, indicating that they played a direct role in restoring calcium pump function. PRAC-E may be a potential candidate for the development of drugs against amyloidosis liver injury.

## Figures and Tables

**Table 1:** Information about 42 ingredients in the TCM–component–target network.

Compounds	Number	Degree Value	Compounds	Number	Degree Value
Albiflorin	**16**	101	Methyl 3,4-dihydroxybenzoate	**6**	8
Paeoniflorin	**17**	101	2-Hydroxy-6-methoxybenzoic acid	**4**	5
Benzoyl paeoniflorin	**18**	98	Homogentisic acid	**12**	5
Catechin	**19**	90	Ethyl trans-caffeate	**39**	5
Gallate	**11**	72	Pelargonic acid	**21**	4
Methyl gallate	**1**	47	3-Hydroxyflavone	**25**	4
Genkwanin	**24**	37	4-Methoxycinnamic acid	**37**	3
Liquiritigenin	**28**	36	3,4-Dihydroxyhydrocinnamic acid	**9**	2
Isorhamnetin	**29**	36	Fructose	**32**	2
Mono(2-ethylhexyl) phthalate	**13**	33	5,7-Dihydroxycoumarin	**36**	2
Pinocembrin	**27**	33	Coniferaldehyde	**38**	2
Sinensetin	**30**	33	3,4-Dimethoxycinnamic acid	**41**	2
Bis(2-ethylhexyl)phthalate	**8**	29	2,6-Dihydroxybenzoic acid	**3**	1
beta-Boswellic acid	**20**	26	4-Hydroxybenzoic acid	**14**	1
Oleic acid	**22**	24	Salicylic acid	**15**	1
Ellagic acid	**7**	19	Citric acid	**23**	1
Epicatechin gallate	**10**	17	Fucose	**31**	1
Ethyl 4-hydroxybenzoate	**2**	16	6,7-Dimethylesculetin	**34**	1
5,7-Dihydroxy-3′,4′,5′-Trimethoxyflavone	**26**	13	4-Methylumbelliferone	**35**	1
Vanillic acid	**5**	10	Ferulate	**40**	1
Glucose	**33**	9	trans-Cinnamaldehyde	**42**	1

**Table 2:** Quantitative determination of SCCs in PRAC-E samples (mg/g).

Components	PRAC-E-1	PRAC-E-2	PRAC-E-3	Mean ± SD (*n* = 3)
Paeoniflorin	14.47	13.45	14.88	14.27 ± 0.74
Albiflorin	7.75	7.01	6.84	7.20 ± 0.49
Gallate	16.22	17.13	17.53	16.96 ± 0.67
Methyl gallate	0.74	0.79	0.80	0.78 ± 0.03
Benzoyl paeoniflorin	5.16	5.74	5.10	5.33 ± 0.35
Catechin	0.08	0.09	0.09	0.09 ± 0.01

## Data Availability

The original contributions presented in this study are included in the article/Supplementary Material. Further inquiries can be directed to the corresponding author.

## Supplementary Materials

The following supporting information can be downloaded at https://www.mdpi.com/article/10.3390/ijms27062582/s1.

## Abbreviations

AA	Amyloid A
AEFs	Amyloid-enhancing factors
ALP	Alkaline phosphatase
ALT	Alanine aminotransferase
Apo-E	Apolipoprotein E
AST	Aspartate aminotransferase
ATP2A1	ATPase sarcoplasmic/endoplasmic reticulum Ca^2+^ transporting 1
CCK-8	Cell counting Kit-8
cGMP	cyclic guanosine monophosphate
CR	Congo red
DEGs	Differential expression genes
ELISA	Enzyme linked immunosorbent assay
HE	Hematoxylin and eosin
HA	Hepatic amyloidosis
IL-6	Interleukin-6
IL-8	Interleukin-8
LYSO	Hen egg-white lysozyme
LYSO-6	Lysozyme amyloid fibrils
PBS	Phosphate-buffered saline
PDE5A	Phosphodiesterase 5A
PKG	Protein kinase G
PRAC-E	Paeoniae Radix Alba Carbonisata extract
SAA	Serum amyloid A
SAP	Serum amyloid P component
SCCs	Six core components
SD	Standard deviation
TCM	Traditional Chinese medicine
TNF-α	Tumor necrosis factor alpha
