# Supplementary material for: Protective Effect of Paeoniae Radix Alba Carbonisata on Hepatic Amyloidosis by Regulating Calcium Homeostasis
Source: Int J Mol Sci. 2026 Mar 11;27(6):2582. doi: 10.3390/ijms27062582 (PMC13026298; doi:10.3390/ijms27062582)
Supplement: Supplementary file 1 [file ijms-27-02582-s001.zip › ijms-4162210-supplementary.pdf]

Supplementary data

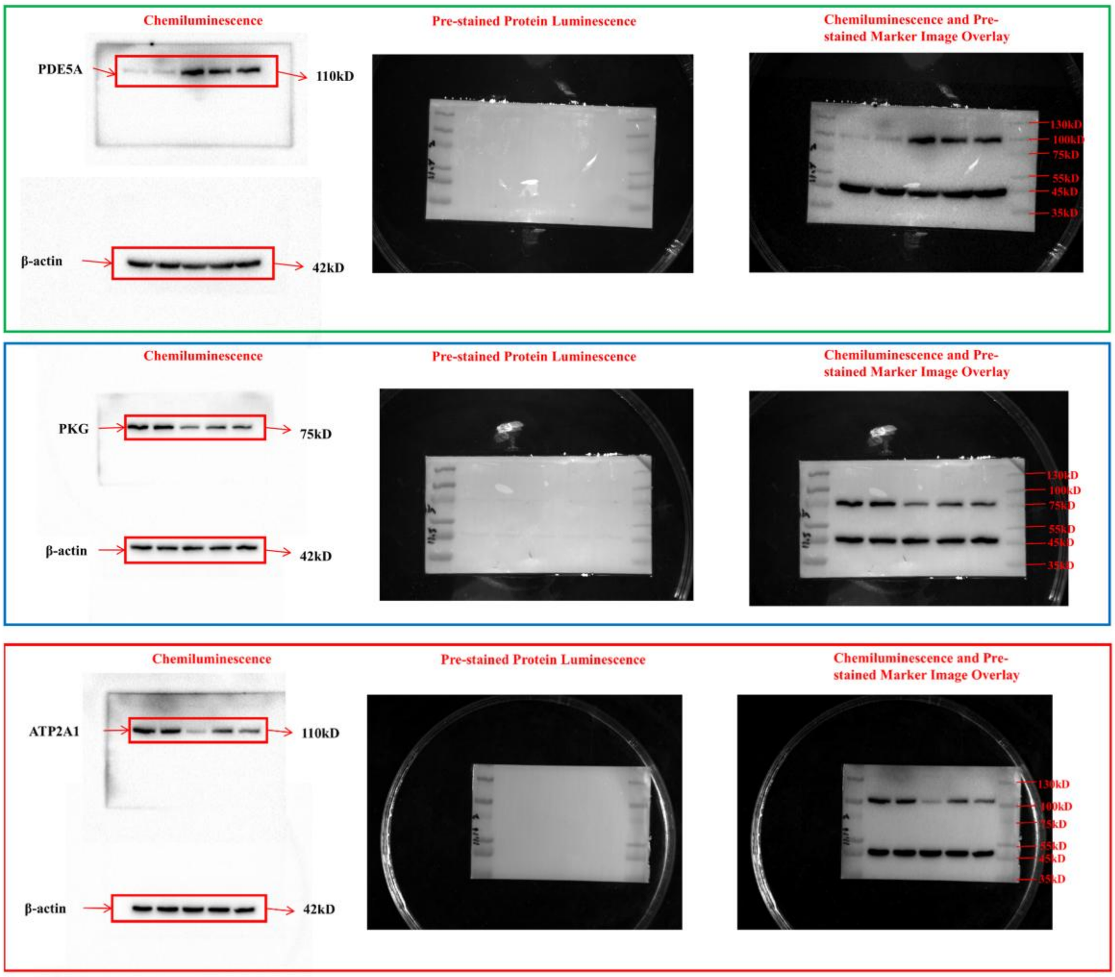

Fig. S1. Representative full Western Blot images showing the expression levels of target proteins.

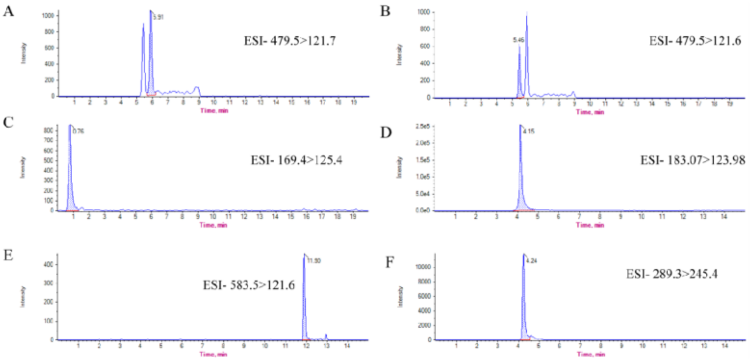

Fig. S2. HPLC-QTRAP-MS/MS (MRM) ion chromatograms of the chemical components.

A: Paeoniflorin; B: Albiflorin; C: Gallic acid; D: Methylgallate; E: Benzoylpaeoniflorin; F: Catechin.

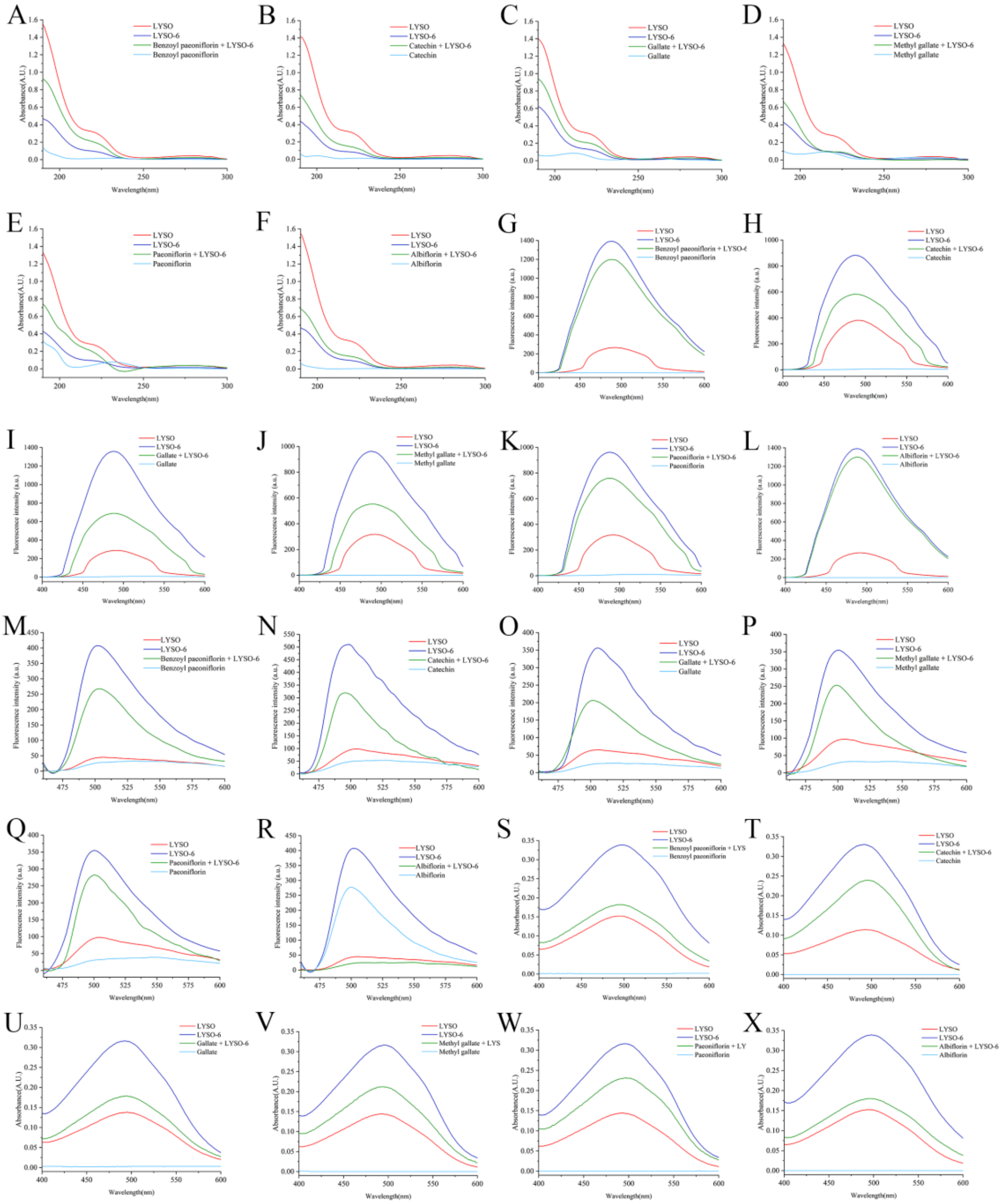

Fig. S3. (A-F) UV-VIS spectra of amyloid fibers incubated under different conditions. (G-L) ANS fluorescence spectra of amyloid fibers incubated under different conditions. (M-R) ThT fluorescence spectra of amyloid fibers incubated under different conditions. (M-R) ThT fluorescence spectra of amyloid fibers incubated under different conditions. (S-X) Visible CR absorption spectra of amyloid fibers incubated under different conditions

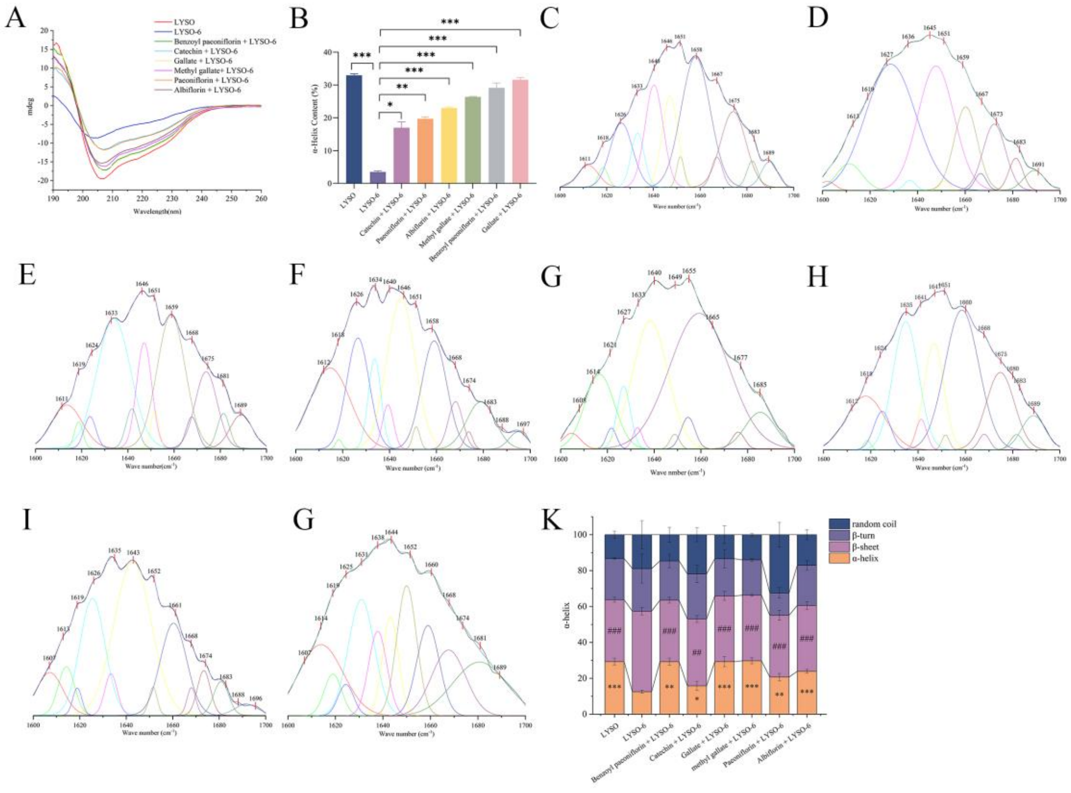

Fig. S4. (A) CD spectra of amyloid fibers incubated under different conditions. (B) α-helix content. (C-G) ATR-FTIR spectra of amyloid fibers incubated under different conditions (C: LYSO; D: LYSO-6; E: Benzoyl paeoniflorin+LYSO-6; F: Catechin+LYSO-6; G: Gallate+LYSO-6; H: Methyl gallate+LYSO-6; I: Paeoniflorin+LYSO-6; J: Albiflorin). (K) Effects of SCCs on secondary structure of amyloid fibers.

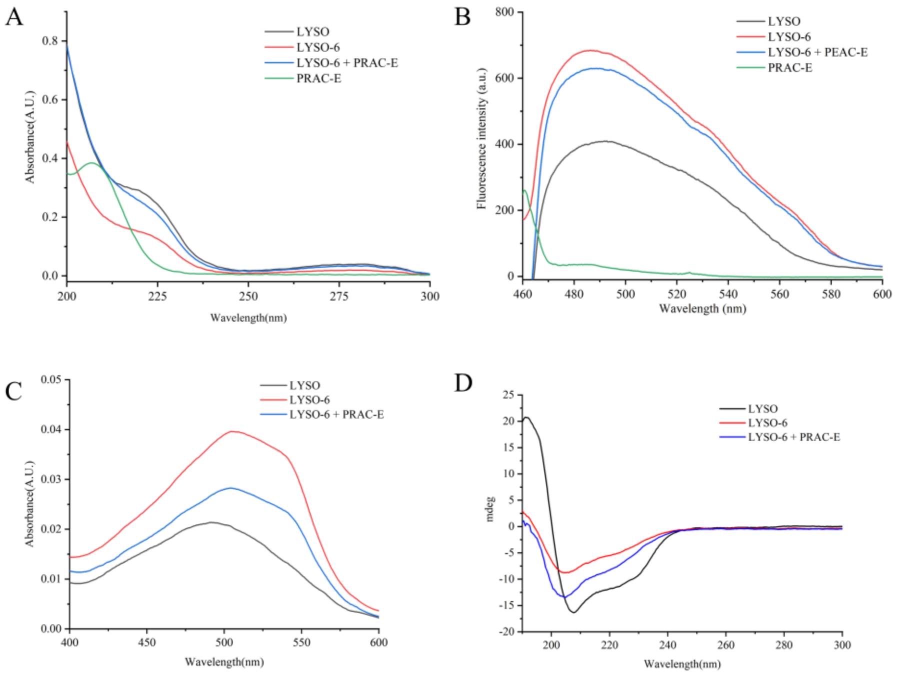

Fig. S5. UV-VIS spectra(A), ThT fluorescence spectra(B), Visible CR absorption spectra(C), and CD spectra(D) of amyloid fibers incubated under different conditions.

Table S1 Qualitative analysis of chemical constituents in C-PRA

No.	t_R(s)	Observed	Formula	Compounds	CAS	Class	Type
1	136.1	169.0134	$C_7H_6O_5$	Gallic acid	149-91-7	Phenolic acids	NEG
2	146.7	183.0289	$C_8H_8O_5$	Methylgallate	99-24-1	Phenolic acids	NEG
3	202.4	165.055	$C_9H_{10}O_3$	4-Ethoxybenzoic acid	619-86-3	Phenolic acids	NEG
4	202.4	165.055	$C_9H_{10}O_3$	Ethyl 3-hydroxybenzoate	7781-98-8	Phenolic acids	NEG
5	202.4	165.055	$C_9H_{10}O_3$	Ethyl 4-hydroxybenzoate	120-47-8	Phenolic acids	NEG
6	88.7	183.0289	$C_8H_8O_5$	3-O-Methylgallic acid	3934-84-7	Phenolic acids	NEG
7	128.6	153.0186	$C_7H_6O_4$	2, 3-Dihydroxybenzoic acid	303-38-8	Phenolic acids	NEG
8	128.6	153.0186	$C_7H_6O_4$	2, 6-Dihydroxybenzoic acid	303-07-1	Phenolic acids	NEG
9	67.8	153.0186	$C_7H_6O_4$	3,5-Dihydroxybenzoic acid	36438	Phenolic acids	NEG
10	155.2	167.034	$C_8H_8O_4$	2-Hydroxy-6-methoxybenzoi-c acid	3147-64-6	Phenolic acids	NEG
11	155.2	167.034	$C_8H_8O_4$	Vanillic acid	121-34-6	Phenolic acids	NEG
12	165.7	167.0339	$C_8H_8O_4$	Methyl 3, 4-dihydroxybenzoate	2150-43-8	Phenolic acids	NEG
13	209.5	300.9971	$C_{14}H_6O_8$	Ellagic acid	476-66-4	Phenolic acids	NEG
14	306.9	391.2811	$C_{24}H_{38}O_4$	DEHP	117-81-7	Phenolic acids	POS
15	307.2	337.2033	$C_{19}H_{30}O_5$	Dodecyl gallate	1166-52-5	Phenolic acids	NEG
16	167.9	151.0394	$C_8H_8O_3$	3-Methoxybenzoic acid	586-38-9	Phenolic acids	NEG
17	145.9	181.0498	$C_9H_{10}O_4$	3, 4-Dihydroxyhydrocinnamic acid	1078-61-1	Phenolic acids	NEG
18	145.9	181.0498	$C_9H_{10}O_4$	Veratric acid	34152	Phenolic acids	NEG
19	196	181.0498	$C_9H_{10}O_4$	Methyl isovanillate	6702-50-7	Phenolic acids	NEG
20	96.7	165.0186	$C_8H_6O_4$	Terephthalic acid	100-21-0	Phenolic acids	NEG

21	176.6	441.0802	$C_{22}H_{18}O_{10}$	Epicatechingallate	1257-08-5	Phenolic acids	NEG
22	64.6	127.0381	$C_6H_6O_3$	Pyrogallol	87-66-1	Phenolic acids	POS
23	174.8	197.0446	$C_9H_{10}O_5$	Ethyl gallate	831-61-8	Phenolic acids	NEG
24	283.9	331.1893	$C_{20}H_{28}O_4$	Carnosic acid	3650-09-7	Phenolic acids	NEG
25	272	205.0843	$C_{12}H_{14}O_4$	Monobutyl phthalate	131-70-4	Phenolic acids	POS
26	272	205.0843	$C_{12}H_{14}O_4$	Neantine	84-66-2	Phenolic acids	POS
27	180.5	123.0446	$C_7H_8O_2$	3-methylolphenol	620-24-6	Phenolic acids	NEG
28	33.7	315.0707	$C_{13}H_{16}O_9$	Gentisic acid 5-O-glucoside	1820-89-9	Phenolic acids	NEG
29	137.8	167.034	$C_8H_8O_4$	3, 4-Dihydroxyphenylacetic acid	102-32-9	Phenolic acids	NEG
30	137.8	167.034	$C_8H_8O_4$	Homogentisic acid	451-13-8	Phenolic acids	NEG
31	145	93.0341	C_6H_6O	Phenol	108-95-2	Phenolic acids	NEG
32	176	123.0431	$C_7H_6O_2$	4-Hydroxybenzaldehyde	123-08-0	Phenolic acids	POS
33	268.1	277.1426	$C_{16}H_{22}O_4$	Mono(2-ethylhexyl) phthalate	4376-20-9	Phenolic acids	NEG
34	191	211.0602	$C_{10}H_{12}O_5$	Propylgallate	121-79-9	Phenolic acids	NEG
35	189.6	137.0237	$C_7H_6O_3$	3-Hydroxybenzoic acid	to check	Phenolic acids	NEG
36	189.6	137.0237	$C_7H_6O_3$	4-Hydroxybenzoic acid	99-96-7	Phenolic acids	NEG
37	189.6	137.0237	$C_7H_6O_3$	Salicylic acid	69-72-7	Phenolic acids	NEG
38	186.3	479.1542	$C_{23}H_{28}O_{11}$	Albiflorin	39011-90-0	Terpenes	NEG
39	186.3	479.1542	$C_{23}H_{28}O_{11}$	Paeoniflorin	23180-57-6	Terpenes	NEG
40	202	493.1689	$C_{24}H_{30}O_{11}$	Harpagoside	19210-12-9	Terpenes	NEG
41	205.6	391.2112	$C_{22}H_{34}O_7$	Forskolin	66575-29-9	Terpenes	NEG
42	28.3	435.1491	$C_{17}H_{26}O_{10}$	Loganin	18524-94-2	Terpenes	NEG
43	280.2	471.3442	$C_{30}H_{48}O_4$	Pomolic acid	13849-91-7	Terpenes	NEG
44	149.8	495.15	$C_{23}H_{28}O_{12}$	6'-(p-Hydroxybenzoyl)mussaenosidic acid	87667-61-6	Terpenes	NEG
45	248.4	167.1071	$C_{10}H_{16}O_2$	(+)-trans-Chrysanthemic acid	4638-92-0	Terpenes	NEG
46	197.9	361.1617	$C_{20}H_{24}O_6$	Triptolide	38748-32-2	Terpenes	POS
47	258.2	487.3411	$C_{30}H_{48}O_5$	Arjunolic acid	465-00-9	Terpenes	NEG
48	258.2	487.3411	$C_{30}H_{48}O_5$	Euscaphic acid	53155-25-2	Terpenes	NEG
49	179.8	495.1481	$C_{23}H_{28}O_{12}$	Oxypaeoniflorin	39011-91-1	Terpenes	NEG
50	305.7	455.3512	$C_{30}H_{48}O_3$	beta-Boswellic acid	631-69-6	Terpenes	NEG
51	172.4	509.1629	$C_{24}H_{28}O_{12}$	Specioside	72514-90-0	Terpenes	POS
52	302.1	311.2936	$C_{20}H_{40}O_2$	Phytanic acid	14721-66-5	Terpenes	NEG
53	177	575.2268	$C_{28}H_{34}N_2O_{11}$	5-Carboxystrictosidine	34371-11-4	Terpenes	POS
54	201.1	443.1331	$C_{23}H_{26}O_{10}$	Lactiflorin	1361049-59-3	Terpenes	NEG
55	220.2	373.2001	$C_{22}H_{32}O_6$	Blinin	125675-09-4	Terpenes	NEG
56	347.5	297.2784	$C_{19}H_{38}O_2$	Pristanic acid	1189-37-3	Terpenes	NEG
57	249.7	317.2113	$C_{20}H_{30}O_3$	Isosteviol	27975-19-5	Terpenes	NEG
58	258.6	489.3535	$C_{30}H_{48}O_5$	Asiatic acid	464-92-6	Terpenes	POS
59	216.1	565.1697	$C_{30}H_{32}O_{12}$	Benzoylpaeoniflorin	38642-49-8	Terpenes	NEG
60	311.6	279.2313	$C_{18}H_{32}O_2$	Linoleic acid	60-33-3	Fatty acids and derivatives	NEG
61	320.8	255.2318	$C_{16}H_{32}O_2$	Palmitic acid	21096	Fatty acids and derivatives	NEG
62	261.1	157.1225	$C_9H_{18}O_2$	Pelargonic acid	112-05-0	Fatty acids and derivatives	NEG
63	306.4	271.2266	$C_{16}H_{32}O_3$	2-Hydroxypalmitic acid	764-67-0	Fatty acids and derivatives	NEG
64	133.2	131.0343	$C_5H_8O_4$	Methylsuccinic acid	498-21-5	Fatty acids and derivatives	NEG
65	322.9	281.2474	$C_{18}H_{34}O_2$	Oleic acid	112-80-1	Fatty acids and derivatives	NEG
66	34.3	115.0394	$C_5H_8O_3$	Levulinic acid	123-76-2	Fatty acids and derivatives	NEG
67	35.6	173.0081	$C_6H_8O_7$	Citric acid	77-92-9	Fatty acids and derivatives	NEG

68	35.6	173.0081	$C_6H_8O_7$	Isocitric acid	320-77-4	Fatty acids and derivatives	NEG
69	27.9	143.0341	$C_6H_{10}O_5$	2-Hydroxyhexanedioic acid	18294-85-4	Fatty acids and derivatives	NEG
70	23.7	105.0187	$C_3H_6O_4$	Glyceric acid	6000-40-4	Fatty acids and derivatives	NEG
71	336.3	283.2626	$C_{18}H_{36}O_2$	Stearic acid	21128	Fatty acids and derivatives	NEG
72	33	103.0395	$C_4H_8O_3$	2-Hydroxybutyric acid	600-15-7	Fatty acids and derivatives	NEG
73	290.4	199.1692	$C_{12}H_{24}O_2$	Dodecanoic acid	143-07-7	Fatty acids and derivatives	NEG
74	40.1	100.0748	$C_5H_{11}NO_2$	5-Aminopentanoic acid	660-88-8	Fatty acids and derivatives	POS
75	306.4	227.2003	$C_{14}H_{28}O_2$	12-Methyltridecanoic acid	2724-57-4	Fatty acids and derivatives	NEG
76	306.4	227.2003	$C_{14}H_{28}O_2$	Myristic acid	544-63-8	Fatty acids and derivatives	NEG
77	197.5	187.0965	$C_9H_{16}O_4$	Azelaic acid	123-99-9	Fatty acids and derivatives	NEG
78	353.7	88.9875	$C_2H_2O_4$	Oxalic acid	144-62-7	Fatty acids and derivatives	NEG
79	240.8	163.0759	$C_{10}H_{12}O_2$	4-Phenylbutanoic acid	1821-12-1	Fatty acids and derivatives	NEG
80	302.1	311.2936	$C_{20}H_{40}O_2$	Arachidic acid	506-30-9	Fatty acids and derivatives	NEG
81	193	305.0655	$C_{15}H_{14}O_7$	(-)-Epigallocatechin	970-74-1	Flavonoids	NEG
82	216.5	285.0753	$C_{16}H_{14}O_5$	3,4-Dihydro-5,7-dihydroxy-4-(4-methoxyphenyl)coumarin	196817-57-9	Flavonoids	NEG
83	212.9	271.0597	$C_{15}H_{12}O_5$	Naringenin chalcone	5071-40-9	Flavonoids	NEG
84	181.9	289.0705	$C_{15}H_{14}O_6$	(+)-Catechin	154-23-4	Flavonoids	NEG
85	212.5	283.0595	$C_{16}H_{12}O_5$	Genkwanin	437-64-9	Flavonoids	NEG
86	29	237.0519	$C_{15}H_{10}O_3$	3-Hydroxyflavone	577-85-5	Flavonoids	NEG
87	199.4	343.0801	$C_{18}H_{16}O_7$	Tricin methyl ether	18103-42-9	Flavonoids	NEG
88	210.5	289.0705	$C_{15}H_{14}O_6$	Epicatechin	490-46-0	Flavonoids	NEG
89	235.4	257.0809	$C_{15}H_{14}O_4$	2',4',6'-Trihydroxydihydrochalcon-e	1088-08-0	Flavonoids	NEG
90	198.2	287.0892	$C_{16}H_{14}O_5$	Sakuranetin	2957-21-3	Flavonoids	POS
91	133.2	122.059	C_7H_7NO	Benzamide	55-21-0	Flavonoids	POS
92	24.1	401.1283	$C_{21}H_{22}O_8$	Hexamethylquercetagetin	1251-84-9	Flavonoids	NEG
93	236.2	255.0653	$C_{15}H_{12}O_4$	Pinocembrin	480-39-7	Flavonoids	NEG
94	178.3	595.1644	$C_{27}H_{32}O_{15}$	Eriodictyol 7-O-neohesperidoside	13241-32-2	Flavonoids	NEG
95	219.4	257.0788	$C_{15}H_{12}O_4$	Liquiritigenin	578-86-9	Flavonoids	POS
96	24.7	543.1288	$C_{30}H_{22}O_{10}$	Rhusflavanone	53060-72-3	Flavonoids	POS
97	184.8	315.0493	$C_{16}H_{12}O_7$	Isorhamnetin	480-19-3	Flavonoids	NEG
98	213.3	273.0732	$C_{15}H_{12}O_5$	(±)-Naringenin	67604-48-2	Flavonoids	POS
99	210.9	371.1114	$C_{20}H_{20}O_7$	Sinensetin	2306-27-6	Flavonoids	NEG
100	154.8	435.1274	$C_{21}H_{24}O_{10}$	Phlorizin	60-81-1	Flavonoids	NEG
101	212.9	147.0445	$C_9H_8O_2$	4-Chromanone	491-37-2	Flavonoids	NEG
102	23.7	341.1069	$C_{12}H_{22}O_{11}$	Trehalose	99-20-7	Saccharide	NEG
103	24.8	365.1029	$C_{12}H_{22}O_{11}$	Maltose	69-79-4	Saccharide	POS
104	24.8	365.1029	$C_{12}H_{22}O_{11}$	Sucrose	57-50-1	Saccharide	POS
105	25.2	323.0969	$C_{12}H_{22}O_{11}$	Isomaltose	499-40-1	Saccharide	NEG
106	25.6	261.0353	$C_6H_{13}O_9P$	Glucose 6-phosphate	56-73-5	Saccharide	POS
107	22.5	261.0365	$C_6H_{15}O_9P$	Glucitol 6-phosphate	20479-58-7	Saccharide	NEG
108	22.5	261.0365	$C_6H_{15}O_9P$	Mannitol 1-phosphate	15806-48-1	Saccharide	NEG
109	25.2	187.0563	$C_6H_{12}O_5$	Fucose	2438-80-4	Saccharide	POS
110	146.3	461.1653	$C_{20}H_{30}O_{12}$	Verbasoside	61548-34-3	Saccharide	NEG
111	143.6	477.1583	$C_{20}H_{30}O_{13}$	Kelampayoside A	87562-76-3	Saccharide	NEG
112	4.7	161.0446	$C_6H_{12}O_6$	Fructose	53188-23-1	Saccharide	NEG
113	4.7	161.0446	$C_6H_{12}O_6$	Glucose	492-62-6	Saccharide	NEG
114	287.2	297.1509	$C_{19}H_{22}O_3$	8-Geranyl-7-hydroxycoumari-n	23660-05-1	Coumarins	NEG

115	212.1	207.0632	$C_{11}H_{10}O_4$	6, 7-Dimethylesculetin	120-08-1	Coumarins	POS
116	19.5	203.0747	$C_{12}H_{10}O_3$	4-Allyloxycoumarin	31005-07-9	Coumarins	POS
117	199.4	193.0479	$C_{10}H_8O_4$	Scopoletin	92-61-5	Coumarins	POS
118	194.9	203.034	$C_{11}H_8O_4$	8-Acetyl-7-hydroxycoumarin	6748-68-1	Coumarins	NEG
119	188.4	175.0391	$C_{10}H_8O_3$	4-Methylumbelliferone	90-33-5	Coumarins	NEG
120	203.7	163.0376	$C_9H_6O_3$	Umbelliferone	93-35-6	Coumarins	POS
121	170.5	207.0633	$C_{11}H_{10}O_4$	7, 8-Dimethoxycoumarin	2445-80-9	Coumarins	POS
122	163	177.0184	$C_9H_6O_4$	5, 7-Dihydroxycoumarin	2732-18-5	Coumarins	NEG
123	206.6	177.0549	$C_{10}H_{10}O_3$	4-Methoxycinnamic acid	830-09-1	Phenylpropanoids	NEG
124	206.6	177.0549	$C_{10}H_{10}O_3$	Coniferaldehyde	20649-42-7	Phenylpropanoids	NEG
125	185.4	359.0754	$C_{18}H_{16}O_8$	Rosmarinic acid	537-15-5	Phenylpropanoids	NEG
126	187.4	223.0599	$C_{11}H_{12}O_5$	3, 5-Dimethoxy-4-hydroxycinna-mic acid	530-59-6	Phenylpropanoids	NEG
127	161.2	207.0652	$C_{11}H_{12}O_4$	Ethyl trans-caffeate	66648-50-8	Phenylpropanoids	NEG
128	172.9	177.053	$C_{10}H_{10}O_4$	Ferulate	537-98-4	Phenylpropanoids	POS
129	323.5	209.0791	$C_{11}H_{12}O_4$	3, 4-Dimethoxycinnamic acid	2316-26-9	Phenylpropanoids	POS
130	241.6	133.0634	C_9H_8O	trans-Cinnamaldehyde	14371-10-9	Phenylpropanoids	POS
131	132.5	139.0379	$C_7H_6O_3$	3, 4-Dihydroxybenzaldehyde	139-85-5	other	POS

* POS-positive ion mode; NEG-negative ion mode

Table S2 Retention time and MS parameters of the chemical components

Compounds	t_R/min	Relative molecular mass	ionization mode	multi-reaction detection	scanning declustering voltage /V	collision energy /eV
Paeoniflorin	5.91	480.16	ESI-	479.50>121.70	-20	-18
Albiflorin	5.46	480.16	ESI-	479.50>121.60	-20	-18
Gallate	0.76	170.02	ESI-	169.40>125.40	-30	-15
Methyl gallate	4.15	184.14	ESI-	183.07>123.98	-30	-22
Benzoyl paeoniflorin	11.90	584.18	ESI-	583.50>121.60	-40	-18
Catechin	4.24	290.07	ESI-	289.30>245.40	-25	-16

Table S3 Regression equation, correlation coefficient (R^2) and linear range of 6 components

Compounds	Regression equation	R^2	Linear range (μg/mL)
Paeoniflorin	y=306.17594x+932.06759	0.99746	0.1564~156.45
Albiflorin	y=509.44679x+510.35116	0.99972	0.1650~165.00
Gallate	y=1283.20387x+29.61658	0.99772	0.1313~131.30
Methyl gallate	y = 198767x+248177	0.99619	0.05450~54.50

Benzoyl paeoniflorin	y=86.20225x-3.45327	0.99233	0.14462~144.62
Catechin	y=2821.73348x+237.97479	0.99569	0.1554~155.4

Table S4 Precision, stability and repeatability

Compounds	RSD（%）		
	Precision	Stability	Repeatability
Paeoniflorin	2.33	1.95	1.24
Albiflorin	1.80	1.50	1.23
Gallate	1.85	1.94	1.31
Methyl gallate	1.34	1.65	1.92
Benzoyl paeoniflorin	1.68	1.98	2.04
Catechin	1.91	1.99	1.82

Table S5 Sample recovery rate

Compounds	Original amount (μg)	Reference substance addition amount (μg)	Measured total amount (μg)	Recovery rate (%)	Average recovery rate (%)	RSD (%)
Paeoniflorin	7.834	9.500	17.472	101.46	100.23	1.62
	7.834	8.000	15.705	98.40		
	7.834	6.500	14.389	100.85		
Albiflorin	3.406	4.000	7.404	99.960	98.96	2.43
	3.406	3.500	6.930	100.70		
	3.406	2.000	5.330	96.21		
Gallate	24.312	29.000	53.240	99.75	99.50	1.94
	24.312	24.000	47.702	97.46		
	24.312	15.000	39.507	101.30		
Methyl gallate	0.660	0.800	1.459	99.87	100.33	0.39
	0.660	0.700	1.364	100.51		
	0.660	0.500	1.163	100.60		
Benzoyl paeoniflorin	2.719	3.200	5.890	99.07	99.88	1.57
	2.719	2.700	5.389	98.87		
	2.719	2.000	4.753	101.69		
Catechin	0.053	0.064	0.117	99.68	101.28	2.46
	0.053	0.053	0.108	104.15		

	0.053	0.042	0.095	100.00
